# Heterogeneous Data Approach on Financial development of Selected African Leading Economies

**DOI:** 10.1016/j.dib.2020.105670

**Published:** 2020-05-11

**Authors:** Ibrahim Farouq, Zunaidah Sulong, Umar Ahmad, Aminu Jakada, Nuraddeen Sambo

**Affiliations:** a University Sultan Zainal Abidin, Malaysia; b World Bank Group

**Keywords:** Westerlund, Dynamic common correlated effect, Heterogeneous, Financial development, Financial globalization uncertainty

## Abstract

The presentation of this data focuses on analysing the dynamic role of economic growth, foreign direct investment and financial globalization uncertainty on financial development of selected leading African economies, spanning the year 1970 to 2018, and the data were obtained from world development indicators and global financial development databases. Second generation econometrics techniques were deployed for the analysis. We began with the descriptive and correlation statistics in order to ascertain the normality of the data. Also, homogeneity and cross-sectional dependency tests were carried out to validate the whether or not the data is heterogeneous and depend upon each other across the series. As well, the [3] co-integration and dynamic common correlated effect [1] and pool mean group [2] estimates were applied to confirm the presence of long-run relationship and their effects on the financial development among the sampled countries.

Specifications table

**Table:** 

Subject	Economics
Specific subject area	Financial economics
Type of data	Tables
How data were acquired	A secondary data extracted from World Development Indicators and global financial development
Data format	Raw Analyzed Filtered Clean
Parameters for data collection	We extracted the relevant data for the analysis from the related data bases
Description of data collection	Quantitative Secondary Data
Data source location	Country: Nigeria, Algeria, Egypt, South-Africa, Ghana, Morocco
Data accessibility	With the article Raw data was deposited in the Mendeley repository as Data for Heterogeneous Data Approach on Financial development of Selected African Leading Economies. doi:10.17632/3779nzm8tz.1 URL:https://data.mendeley.com/datasets/3779nzm8tz/draft?a=712517bd-1756-4864-8f7e-9e29e90c7559

## Value of the data

• The data article aimed at examining the effects of macroeconomic variables on financial development. Thus, It's useful as it combines five components of financial development through the use of principal component analysis for the financial development index as not always been the case in the past. Thus, measuring the impact of these determinants on the development of the financial sectors based on the aggregate performance will surely give an insight to the policy makers.
• Indeed, the respective governments of the used economies will find the data helpful, due to the fact that less attention was paid to the leading economies with regards to financial development data, more especially the uncertainty part of the financial globalization, given the continues integration of these economies with the rest of the world. Moreover, investors will as well benefit from the data in identifying market-friendly environment.
• The data have proven that the parameters are heterogeneous and depend upon each order across the countries, which means, as shown by this data other advanced methods of analysis could be used for further empirical data analysis.
• Its uniqueness in accommodating second generation econometrics techniques

## 1 Data Description

The data comprises of 288 year-country observation generated from the world development indicator and global financial development data bases. The data covers forty-eight (48) years from 1970 until 2018. We analyses the direct relationships between economic growth, foreign direct investments and financial globalization uncertainty on financial development in six selected leading African economies using heterogeneous econometrics approach. Tables 1 and 2 describes the normality of the data, while Table 3 and 4 validates the ability of the data to run heterogeneous techniques. Also, table 5 shows the co-integration result and table 6 indicates the short and long-run estimates. (Table 7)

**Table 1:** Summary Statistics

Variables	Mean	Standard deviation	Skewness	Kurtosis	Jarque-Bera
FD_it_	5.44E-09	1.000	−0.983	6.001	157.767 (0.000)
GDP_it_	1.364274	0.803240	−1.998509	12.00122	1188.228* (0.000)
FDI_it_	310.381	2.763	−4.154	25.599	7102.102* (0.000)
FGU_it_	−7.48E-08	1.068618	−0.448850	9.581368	540.4734* (0.000)

**Table 2:** Correlation matrix

Variables	LNFD_it_	LNGDP_it_	LNFDI_it_	LNFGU_it_
LNFD_it_	**1.000**			
LNGDP_it_	0.256* (0.000)	**1.000**		
LNFDI_it_	0.028 (0.625)	−0.131 (0.023)	**1.000**	
LNFGU_it_	0.039 (0.500)	0.096 (0.098)	−0.123** (0.034)	**1.000**

Notes: * and ** significant at 1 and 5% levels. Figures in () denote p-values

**Table 3:** Cross-sectional Dependence Tests

Variables	Pesaran CD test	Pesaran LMCD test	Breush-Pagan (LM) test
LNFD_it_	7.430* (0.000)	10.491* (0.000)	72.465* (0.000)
LNGDP_it_	2.494** (0.012)	6.811* (0.000)	52.309* (0.000)
LNFDI_it_	14.887* (0.000)	42.565* (0.000)	248.142* (0.000)
LNFGU_it_	2.665* (0.007)	2.450** (0.014)	28.424** (0.019)

Note: * and ** significant at 1 and 5% levels. Figures in () denote *p*-values. Pesaran (2004) CD test takes cross-independence as the null, and the p-values are for the one-sided test based on the normal distribution.

**Table 4:** Slope Homogeneity Tests

Group	Test	Statistic	P-value
	Delta	2.562*	0.010
	Adjusted Delta	2.707*	0.007

*shows statistical significance at % level

**Table 5:** Panel Unit Root Test

Variables	CIPS		CADF	
	At level	At first different	At level	At the first diff
*LNFD_it_*	−4.916* (0.000)	−6.420* (0.000)	−4.520* (0.000)	−6.253* (0.000)
*LNGDP_it_*	−5.154* (0.000)	−6.234* (0.000)	−3.902 (0.000)	−6.092* (0.000)
*LNFDI_it_*	−4.645* (0.000)	−6.265* (0.000)	−4.586 (0.000)	−5.726* (0.000)
*LNFGU_it_*	−5.774* (0.000)	−6.420* (0.000)	−4.433* (0.004)	−6.327 (0.000)

Notes: *, ** and *** Denotes rejection of the null hypothesis at 1% and 5% and 10% significance level.

**Table 6:** Summary Results of Heterogeneous Co-integration Tests

	Statistic	With Trend		Without Trend	
		Value	*p*-value	Value	*p*-value
Westerlund	*G*_t_	−4.785*	0.000	−4.544*	0.000
	*G*_a_	−27.234*	0.017	−28.168*	0.000
	*P*_t_	−10.936*	0.000	−10.745*	0.000
	*P*_a_	−25.906*	0.003	−27.049*	0.000

Note: * and ** denote rejection of the null hypothesis of no cointegration at 1 and 5% levels of significance

**Table 7:** Dynamic Common Correlated Effect and Pooled Mean Group Estimate

Short-run Estimate									
DCCE							PMG		
Variables	Coefficient		z		P-value		Coefficient	Z	P-value
LGDP_it_	0.163 (0.079)		2.05		0.040		0.089 (0.037)	2.37	0.018
LFDI_it_	−0.021 (0.008)		−2.57		0.010		−0.020 (0.008)	−2.41	0.016
LFGU_it_	−0.036 (0 .050)		−0.72		0.474		−0.047 (0.032)	−1.48	0.140
ECT							−0.555 (0.086)	−6.41	0.000
**Long-run Estimate**									
LGDP_it_		0.543 (0.212)		2.56		0.011	0.189 (0.092)	2.06	0.040
LFDI_it_		−0.081 (0.386)		−2.12		0.034	0.007 (0.002)	2.92	0.004
LFGU_it_		0.114 (0.188)		0.61		0.545	0.062 (0.109)	0.57	0.566
		Cross-section		6					
		Observations		288					
		R-squared		0.49					
		Adj. R-squared		0.41					
		F-Stat (p)		5.72 (0.000)					

Evidence based on the data analysis presented indicates that the present data is heterogeneous in nature and therefore affirmed the use of second generation method of analysis. Also, as expected the pool mean group estimate records that it will take 55 percent speed for the model to return to equilibrium at the long-run in the case of any deviation. Moreover, economic growth is positively and statistically significant towards financial development, whereas, foreign direct investment shows a negative but significant to the development of the financial sector of the sampled countries. But, financial globalization uncertainty records no significant effect on financial development.

## 2 Experimental Design, Materials, and Methods

In this data article, we deployed a dataset extracted from the secondary source databases as mentioned earlier. All preliminary tests were conducted in order to validate the data and have a clear picture of what the data be. The tests ranging from the descriptive statistics to ascertain the normality of the data, the correlation matrix to test for multi-collinearity among the regressors. Besides, cross-sectional dependency test was also carried out to see whether the data is dependent across the series or not, as well as the homogeneity test to determine if the variables are heterogeneous among the countries. These last two tests are necessary for cross-sectional data, so that one can easily identify the appropriate methods to use in order to avoid having bias report. After which, the unit root, co-integration and estimation tests applied were all heterogeneous techniques as supported by the data.
